# Fishing for answers: is oxidation of fish oil supplements a problem?

**DOI:** 10.1017/jns.2015.26

**Published:** 2015-11-23

**Authors:** David Cameron-Smith, Benjamin B. Albert, Wayne S. Cutfield

**Affiliations:** Liggins Institute, University of Auckland, Auckland, New Zealand

Fish oils, rich in *n*-3 PUFA, have become one of the most popular dietary supplements worldwide with millions of regular consumers^(^^1^^)^. Sales in the USA alone exceed US$ 1 billion annually^(^^2^^)^. There is a broad range of benefits claimed for *n*-3 fish oils including: prevention of CVD^(^^3^^)^, reduced cognitive decline^(^^4^^)^, and the improved management of inflammatory diseases (arthritis, inflammatory bowel disease and asthma)^(^^5^^)^. However, a series of recent studies has not demonstrated significant benefits, particularly regarding the secondary prevention of CVD^(^^6^^,^^7^^)^.

*n*-3 PUFA are highly prone to oxidative degradation, making fish oils one of the most labile supplements sold to consumers. Recently in the *Journal of Nutritional Science*, Jackowski *et al.* evaluated primary and secondary oxidation in all of the *n*-3 fish oils available over the counter in retail stores in Canada^(^^8^^)^. A total of 171 supplements from forty-nine brands were assessed, with 50 % exceeding voluntary limits for at least one measure of oxidation, and 39 % exceeding the international voluntary safety recommendations for total oxidation (TOTOX) value. These findings are not unique to Canada. In the USA, 27 % of products tested were found to have more than twice the recommended levels of lipid peroxides^(^^9^^)^, while in South Africa^(^^10^^)^ and New Zealand^(^^11^^)^ more than 80 % of supplements tested exceeded recommended levels.

The oxidation of *n*-3 PUFA is complex, and the degree and rate of oxidation of fish oil are influenced by many factors, including fatty acid composition, exposure to O_2_ and light, temperature, antioxidant content, and the presence of water and heavy metals^(^^12^^)^. The initial stage of oxidation of fish oils leads to increased levels of hydroperoxides, which decompose into a variety of radicals^(^^12^^)^. These react with unoxidised PUFA to form additional hydroperoxides, while also breaking down to form a wide range of possible secondary oxidation products such as volatile ketones and alcohols. These are strongly linked to the rancid smells and off flavours^(^^12^^,^^13^^)^.

While oxidation leads to a complex array of primary and secondary oxidation products, the degree of oxidation can be characterised by just two industry-standard assays. The peroxide value (PV) provides a quantitative measure of hydroperoxide levels. The most common method to estimate secondary oxidation is the calculation of the anisidine value (AV), which provides a measurement of aldehydic compounds (predominately 2-alkenals and 2,4-alkadienals). By measuring both PV and AV, primary and secondary oxidation can be characterised, enabling an overall assessment of the degree of oxidation. This is reflected in the TOTOX value (=2PV + AV)^(^^14^^)^. A number of authorities have published maximum limits of oxidation in fish oils^(^^15^^–^^17^^)^, including the Global Organization for EPA and DHA Omega-3s (GOED), a trade organisation^(^^18^^)^. The maximum recommended limits are: PV 5 mEq/kg, AV 20, and TOTOX 26.

It is not surprising that many retail fish oil products are oxidised to varying degrees, when one considers the complex process from ocean catch through to the final consumer product. The major sources of fish oil are small pelagic fishes, caught off the coast of Peru and Chile^(^^19^^)^. Each catch is transported on a fishing vessel to shore, where it is then processed by fractionation into fish meal and crude fish oil. The oil produced is stored in large tanks before being shipped on for further refining, particularly to China. This refining process typically involves several steps, notably including repeated heating at high temperatures. The last stage of refinement is deodorisation to remove NEFA, aldehydes and ketones, which are responsible for the undesirable taste and rancidity of oxidised oils^(^^15^^)^. Less than 25 % of the total crude fish oil supply is destined for human consumption and undergoes additional refinement and deodorisation. The remainder is predominantly used in the aquaculture industries^(^^19^^)^. As a result, fish oil supplements are just one small part of an international commodity trade, where early steps in processing are not specific for supplement production and the catch, isolation, purification and manufacture of oil all occur well removed from the final consumer market. Therefore, there is limited opportunity for the consumer to link the source, the age of the product, the extent and process of refinement with the marketed and packaged final consumer product.

The end result is that consumers are at risk of purchasing an oxidised supplement, for which there is little tangible information on the packaging to provide details of the oil's original source, age and levels of refinement. The levels of oxidation now described in four independent studies since 2012 (analysing 260 *n*-3 PUFA products) suggest that the general public is consuming oxidised products exceeding voluntary industry-standard levels. Importantly, the biological effects and health consequences of consuming oxidised fish oil supplements are not yet established. In 2010, the European Food Standards Authority (EFSA) panel on biological hazards presented a scientific opinion on fish oil for human consumption^(^^15^^)^, concluding that ‘information on the level of oxidation of fish oil (as measured by peroxide and anisidine values) and related toxicological effects in humans is lacking’.

Of note, it must also be recognised that *n*-3 PUFA supplements used in previous clinical trials may have been oxidised. It is therefore possible that the trial literature may have been significantly confounded by the use of oxidised oils. As a result, there should be independent analyses of fish oils adopted in clinical trials, and their oxidative state should be reported in future studies.

Jackowski *et al*.^(^^8^^)^ and similar studies highlight a number of important issues that need to be resolved regarding fish oil supplements. There is pressing need for research that can establish the effects of oxidised oils on human health and the safe limits of oxidation for human consumption. Further, greater monitoring is required to ensure that over-the-counter products meet recommended limits.
